# Albuminous Urine in Puerperal Convulsions

**Published:** 1864-02

**Authors:** F. H. Ramsbotham


Art. IV.—*Albuminous Urine in Puerperal Convulsions.* By F. H. Ramsbotham, M. D.

This eminent accoucheur, in a recent communication to the Medical Times and Gazette, says: "Shortly before this time, October, 1843, my attention was directed to the fact of the urine in women who became the subjects of convulsions in labor containing more or less albumen, sometimes in considerable quantity; and I have found in my own practice this to be the case in about every five cases out of six where I have been able to make the observation. But, as the patient is recovering, it gradually becomes less and less, and it has almost entirely disappeared at the end of twelve or fourteen days, sometimes earlier, though occasionally it persists for a longer time. Some pathologists imagine that the presence of albumen, under such circumstances, indicates granular disease of the kidneys, and therefore attribute the convulsive seizures to those bodies having become the seat of that particular organic change. On the contrary, we are told by M. Blot and others that the excretion of albumen by the kidneys in small quantity is not an unusual occurrence in pregnant women. If organic disease of the kidneys was present in all cases of puerperal convulsions, how could we account for the disappearance of the albumen so soon after delivery—we should expect it to remain permanently. The following is the explanation which has occurred to my own mind: we know that the fibrine is in excess in the blood of pregnant women, we have reason to believe that the albumen is in excess also; and from the observations of M. Blot, that a part of that access is got rid of by the kidneys, we may easily imagine that if there is a much greater quantity formed than healthy blood ought to contain, the superfluous albumen may excite the apoplectic seizure which is the occasion of the fits; and that thus the connexion between albuminous urine and puerperal convulsions as cause and effect is manifest.

